# In memoriam: Susan Abmayr (1956–2019) – “What do we do? Whatever it takes!”

**DOI:** 10.1186/s13395-019-0215-0

**Published:** 2019-11-27

**Authors:** Erika R. Geisbrecht, Mary K. Baylies

**Affiliations:** 1 Department of Biochemistry and Molecular Biophysics, Kansas State University, 141 Chalmers Hall, Manhattan, KS 66506 USA; 2 Program in Developmental Biology, 430 East 67th Street, Box 310, New York, NY 10065 USA



We are saddened to announce that Susan Abmayr, noted pioneer in *Drosophila* myogenesis, passed away suddenly on Thursday, July 18, 2019. Susan was born on March 13, 1956 in Pittsburgh, PA. She obtained her bachelor’s degree in Biological Sciences and Economics from Carnegie Mellon University in 1978 and completed her graduate training in 1987 under the mentorship of Robert G. Roeder, Ph.D. at Rockefeller University studying basic mechanisms of transcription. During her time in the Roeder lab, Susan met her husband and longtime collaborator, Jerry Workman, Ph.D. She performed her postdoctoral work with Tom Maniatis, Ph.D. in the Department of Biochemistry and Molecular Biology at Harvard University. Susan started her independent research career in the Department of Biochemistry and Molecular Biology at Penn State University and was promoted to Associate Professor in 1998. In 2003, Susan moved to the Stowers Institute for Medical Research in Kansas City as an Associate Investigator. She received a secondary appointment at the University of Kansas School of Medicine in 2004.

Susan’s contributions to the fields of transcription and myogenesis resulted in over 70 publications. Her scientific career and introduction to *Drosophila* as a model organism began in Sarah C. R. Elgin’s laboratory at Harvard University where she worked as a technician before starting graduate school. It was in the Elgin lab where Susan became familiar with chromatin organization and gene expression and forged life-long connections with fellow Elgin lab members [1–3]. Once in graduate school, she continued to pursue research questions related to transcription in the Roeder lab, with an emphasis on understanding transcriptional initiation by TFIID binding to promoter sequences [4–7].

Supported by a Damon Runyon-Walter Winchell Cancer Research Fund Post-Doctoral fellowship in the Maniatis lab, Susan was at the forefront in establishing *Drosophila* as a myogenic model. Only very few labs, among them Michael Bate’s lab in Cambridge UK, were using *Drosophila* to study muscle development at that time [8]. Susan sought to bring her expertise in transcription to the fly. In 1989, Harold Weintraub’s group reported the isolation of mouse MyoD, a master regulatory gene for myogenic determination [9]. When injected into non-muscle cell types, such as melanoma, neuroblastoma, liver, and adipocytes, MyoD transformed them into muscle. Capitalizing on the relative simplicity and ease of fly genetics, Susan merged her background in transcription with fly biology to uncover a *Drosophila* homolog of MyoD. In collaboration with her colleague Alan Michelson, they used the helix-loop-helix (HLH) regions of mouse MyoD and rat Myogenin as hybridization probes to screen a *Drosophila* genomic library. The identification of this fly MyoD protein, dubbed ‘Nautilus’ after the weight machine at the gym [10], broke open the embryonic myogenesis field in *Drosophila* and subsequently paved the way for the discovery of vertebrate Myocyte-specific Enhancer Factor 2, or Mef2 by Susan and other labs [11–16]. The absence of Mef2 results in a lack of muscle tissue. Without differentiation of naïve embryonic cells into myoblasts in these mutant embryos, the development of muscles fails.

At a time when the central focus of *Drosophila* studies was either on patterning the embryonic epidermis or on the establishment of the nervous system [17, 18], the advantages of using this stage of development to understand myogenesis became readily apparent. Muscle cell fate specification, myoblast fusion, myotube guidance, and attachment all occur in the relatively short time frame of ~ 10 h [19–23]. Moreover, the genetic tools and numerous reagents to follow individual proteins both in fixed and live tissue have allowed for a detailed dissection of myogenic events that are not possible in cell culture or mammalian models. One great example of exploiting this model system has been the use of genetic screens to identify molecules essential for myoblast fusion, which has been much of the focus of Susan’s research career.

The myogenesis field was mammalian focused in the late 1980s and early 1990s. *Drosophila* as an experimental system to study myogenesis was considered somewhat on the fringe at this time, yet this gave Susan a unique niche when she started her independent laboratory at Penn State University. Susan’s early years could best be classified as the years of discovery. While trying to make mutations in *nautilus*, Susan’s lab identified two genes required for the fusion of myoblasts to generate multinucleated myofibers. The first was *sticks and stones (sns)* which encodes for a transmembrane protein that is part of the immunoglobulin (Ig) superfamily [24]. Sns is present on the surface of the fusion competent myoblasts (FCMs) [24, 25]. There it interacts at the sites of fusion with the Ig domain family member Dumbfounded (Duf) which is present on founder cells (FCs), or seed myoblasts, that give rise to an eventual syncytial muscle cell [25, 26]. Embryos that lack Sns have an abundance of unfused myoblasts that fail to form the stereotypical, multinucleated myofibers present in wild-type embryos [24, 27, 28]. The second gene uncovered was *myoblast city (mbc)* [29]*.* The Mbc protein is a cytoplasmic protein that functions with the GTPase Rac to regulate the actin cytoskeleton. A quote from Susan in a 1994 Penn State publication [30] noted the novelty of her approach, “*Not many people have looked at developing muscle in a fly embryo. We’re some of the first people in the country to start identifying these kinds of genetic defects.”*

Susan’s foresight to use the fly system to identify conserved factors required for myogenesis quickly drew others to the field which resulted in the initiation of multiple genetic screens in other labs to uncover novel fusion mutants [31–34]. It was around this time in 2003 when Susan and Jerry moved their labs to the Stowers Institute. A major focus of the lab continued to be the identification of new players required in muscle development using the state-of-the-art technologies that Susan was so willing to incorporate. Expansion into proteomic approaches identified Elmo/Ced-12 as an obligate binding partner of Mbc to modulate actin cytoskeletal activity at the site of fusion [35]. More importantly, the discovery of these early genes transitioned the field into characterizing the cellular events that govern the myoblast fusion process. This transition also brought with it Susan’s development of timelapse imaging approaches that were being pioneered in the field [36]. Current models derived from the work in Susan’s lab and others show that FCMs must migrate and adhere to existing FCs. Cell adhesion mediated by Sns in the FCM and Duf in the FC relay signaling information through the MBC-ELMO-Rac pathway to mediate actin dynamics. Actin foci formation is dependent on the Formin, Diaphanous and, most notably, the Arp2/3 complex, which nucleates and drives actin-based polymerization [37–42]. The transient F-actin foci that is formed and resolved at the site of each membrane fusion event is accompanied by protrusions that induce membrane destabilization, pore formation, and ultimately fusion of the opposing lipid bilayers [43, 44]. Notably, many of the genes discovered in the *Drosophila* system by Susan and other fly labs have been later proven to be required for the fusion of vertebrate muscles [45].

Beyond her research achievements, Susan was well respected for her contributions in the *Drosophila* and myogenesis communities. She acted as the Heartland representative on the *Drosophila* Board, served as a grant reviewer for the National Institutes of Health (NIH) and the National Science Foundation (NSF), and helped organize a Frontiers in Myogenesis meeting in 2006. Susan defined what it meant to be an educator at many levels. At the forefront of her priorities was the training of graduate students. She was a passionate lecturer and a firm believer in the power and rigor of genetic approaches. After moving the lab from Penn State to the Stowers Institute, she was actively involved in graduate student recruiting and admissions through the University of Kansas Medical School (KUMC) graduate program for over 14 years. Susan’s mentoring also extended beyond her students: for example, she was an invaluable colleague on study panels, offering advice and counselling to new panel members on how to navigate the proper grant review.

Susan’s first love of transcription never wavered as she maintained a long-standing collaboration in the chromatin field with her husband, Jerry Workman. Her expertise in *Drosophila* genetics added an innovative angle to Jerry’s work that culminated in over 35 co-authored papers, primarily understanding the tissue-specific roles of Spt-Ada-Gcn5-acetyltransferase (SAGA) complexes during development. Outside of the lab, Susan and Jerry enjoyed extensive traveling and visiting with family. She also loved gardening, audiobooks, and Billy Holliday music. A late passion was volunteering through Uplift, a Kansas City based organization devoted to serving the homeless.

Every mentor, even subconsciously, instills scientific traits in their trainees that become ingrained and get passed onto future generations of researchers. Two words that embodied Susan’s approach to science were ‘rigor’ and ‘persistence.’ Expectations required accuracy and precision. Repetition assured both. It was rare for a project to be put on the sidelines. Every piece of information would eventually make sense with more experimentation. She would frequently wait in the lab until late in the evening to see the latest scientific result and never wavered in her commitment to student success, whether that be assisting with additional studying to pass a Ph.D. qualifying exam or making numerous revisions on a Ph.D. thesis. She will be missed by friends, colleagues, and the numerous trainees she mentored.
